# The B-MaP-C study: Breast cancer management pathways during the COVID-19 pandemic. Study protocol

**DOI:** 10.1016/j.isjp.2020.07.003

**Published:** 2020-07-29

**Authors:** Alona Courtney, Rachel O'Connell, Tim Rattay, Baek Kim, Ramsey I. Cutress, Cliona C. Kirwan, Ashu Gandhi, Patricia Fairbrother, Nisha Sharma, Christopher W.J. Cartlidge, Kieran Horgan, Stuart A. McIntosh, Daniel R. Leff, Raghavan Vidya, Shelley Potter, Chris Holcombe, Ellen Copson, Charlotte E. Coles, Rajiv V. Dave

**Affiliations:** a Department of Surgery and Cancer, Imperial College London, UK; b Department of Breast Surgery, The Royal Marsden NHS Foundation Trust, Downs Road, Sutton, Surrey SM2 5PT, UK; c Department of Cancer Studies, Clinical Sciences Building, University of Leicester, Leicester LE2 2LX, UK; d Department of Breast Surgery, St. James’s University Hospital, Leeds LS9 7TF, UK; e University of Southampton and University Hospital Southampton, Tremona Road, Southampton SO16 6YD UK; f The Nightingale Breast Cancer Centre, Wythenshawe Hospital, Manchester M23 9LT, UK; g Division of Cancer Sciences, School of Medical Sciences, Faculty of Biology, Medicine and Health, University of Manchester, Oglesby Cancer Research Building, Manchester Cancer Research Centre, Wilmslow Road, Manchester M20 4BX, UK; h Trustee, Independent Cancer Patients Voice, UK; i Breast Unit, Level 1 Chancellor Wing, St James’s Hospital, Leeds LS9 7TF, UK; j Queen Margaret Hospital, Dunfermline, Whitefield Rd, Dunfermline KY12 0SU, UK; k Patrick G Johnston Centre for Cancer Research, Queen’s University Belfast, 97 Lisburn Road, Belfast BT9 7AE, UK; l The Royal Wolverhampton NHS Trust, Wolverhampton Road, Wolverhampton WV10 0QP, UK; m Bristol Centre for Surgical Research, Population Health Sciences, Bristol Medical School, Canynge Hall, Whatley Road, Bristol BS8 2PS UK; n Linda McCartney Centre, Royal Liverpool and Broadgreen University Hospital, Prescot Street, Liverpool L7 8XP, UK; o Department of Oncology, University of Cambridge, UK

**Keywords:** Breast cancer, COVID-19, Treatment, Outcomes, Audit, Standard treatment

## Abstract

**Introduction:**

Approximately 55,000 women in the United Kingdom are diagnosed with new breast cancer annually. Since emerging in December 2019, SARS-CoV-2 (coronavirus disease 2019, COVID-19) has become a global pandemic, affecting healthcare delivery worldwide. In response to the pandemic, multiple guidelines were issued to assist with rationalising breast cancer care. The primary aim of the B-MaP-C study is to audit and describe breast cancer management of patients newly diagnosed with breast cancer during the COVID-19 pandemic against pre-COVID-19 management practice in the UK. The implications of changes to management will be determined and the impact of a COVID-19 diagnosis on the patient’s breast cancer management will be determined.

**Methods and analysis:**

This is a multi-centre collaborative audit of consecutive breast cancer patients undergoing treatment decisions during the acute and recovery phases of the COVID-19 pandemic. All patients with newly diagnosed primary breast cancer, whose treatment was decided in a multidisciplinary meeting from the 16th March 2020, are eligible for inclusion.

**Ethics and dissemination:**

As this is an audit ethical approval is not required. Each participating centre is required to register the study locally and obtain local governance approvals prior to commencement of data collection. Local audit data will be available to individual participating units for governance purposes. The results of the data analysis will be submitted for publication, as well as disseminated via the ABS newsletter and a webinar. All data will be presented at national and international conferences, circumstances permitting.

**Registration details:**

Each participating centre received local governance audit registration.

## 1 Background

Approximately 55 000 women in the United Kingdom are diagnosed with new breast cancer each year [1]. Multi-modal treatment involves a combination of surgery and systemic therapy, which often involve multiple hospital visits or admissions, increasing the potential risk of exposure to COVID-19. The first case of the novel coronavirus SARS-CoV-2 (coronavirus disease 2019, COVID-19) was diagnosed in the UK on the 23rd January 2020 [2], with the first death recorded on the 5th March [3], [4]. As of 5th June 2020, COVID-19 has resulted in 6,515,796 confirmed cases and 387,298 deaths worldwide since its emergence in December 2019 [5]. Globally, the COVID-19 pandemic has led to repercussions in healthcare delivery, including alterations in cancer care. In the UK, the Association of Breast Surgery (ABS) published a guidance statement, outlining the temporary changes required to the work-up of patients with breast symptoms and the surgical management of patients with a new diagnosis of breast cancer [6]. The guidelines were intended to help breast units rationalise delivery of breast services whilst healthcare resources were limited and when hospitals were considered a high risk environment for many vulnerable patients.

In summary, the ABS advised the following changes to be implemented [6]:

1. If operating theatre capacity was limited, patients should be prioritized for surgery in the following order: oestrogen receptor (ER) negative cancers, human epidermal growth factor receptor 2-positive (HER2+) cancers, pre-menopausal ER+ cancers, post-menopausal ER+ cancers, high grade ductal carcinoma in situ (DCIS), intermediate or low grade DCIS. In case of insufficient theatre capacity, post-menopausal ER+ patients could be commenced on endocrine therapy.
2. Neoadjuvant chemotherapy should only to be offered to patients with inoperable disease and should not be used to downstage from mastectomy to breast conserving surgery (BCS) or to perform axillary conservation in patients with ER− or HER2+ disease. This is to avoid a potential delay to definitive surgical treatment of the primary cancer.
3. All immediate breast reconstruction (IBR) was suspended, with delayed reconstruction to be offered at a later date once the service returned to normal to minimise surgical complexity, length of stay, complication risks, therefore reducing the risk of developing COVID-19.
4. Genomic testing on the core biopsy should be considered in all grade 3 or node positive ER+ patients [6]. Patients with a high recurrence score should be advised to have surgery as they would ordinarily need adjuvant chemotherapy.

The Royal College of Radiologists (RCR) produced guidelines on the rationalisation of radiotherapy (RT) for breast cancer patients during the pandemic [7] subsequently supported by International guidelines. These were in part based on the published results of the Fast Forward trial, which demonstrated non-inferiority for local recurrence for 5 fractions of adjuvant radiotherapy compared to the UK standard of care 15 fraction [8], [9].

The guidelines recommended the following options be considered and discussed with the patient for shared decision-making: Radiotherapy omission for patients over 65 years (or younger comorbid patients) with invasive breast cancer of 30 mm or less, provided they fulfilled the following criteria: clear margins, grade (G) 1–2, ER+, HER2- and node negative commencing endocrine therapy. RT could be delivered in 5 fractions for node negative disease. Boost RT could be offered only to patients under 40 years or those over 40 with high risk of relapse. Nodal RT could be omitted in the following cases: 1) post-menopausal women undergoing whole breast RT after sentinel lymph node biopsy; 2) primary surgery for T1, ER+, HER2- G1-2 tumours with 1–2 macrometastases [7].

The guidelines also highlight that pre-operative breast radiotherapy could be used as an emergency bridging measure in patients where surgery was postponed during the COVID-19 pandemic and further pre-operative systemic therapy was not possible. These included patients with a new diagnosis of invasive breast cancer or patients completing neoadjuvant therapy, where systemic therapy was not appropriate, or patients with disease progression despite use of systemic therapies [10].

In line with the national guidelines [11], the Cancer Core Europe (CCE) consortium published general consensus measures on the re-structuring of service delivery during the COVID-19 pandemic [12]. They recommended to record and analyse the impact of treatment alterations during the pandemic on the cancer specific patient outcomes [12]. Pardo et al. published a suggested approach to the management of breast cancer patients during different stages of the pandemic, based on their local experience [13]. Several other publications reported the outcomes of COVID-19 disease in cancer patients [14], [15], [16], although none of these papers focused specifically on the outcomes of the breast cancer patients [17].

### 1.1 Aims and objectives

The primary aim of the B-MaP-C study is to audit and describe breast cancer management during the COVID-19 pandemic against standard pre-COVID-19 management practice. In addition, the impact of a COVID-19 diagnosis on the patient’s breast cancer management will be determined.

The short-term audit objectives are to determine the impact of the COVID-altered management on the patient outcomes, including:

1. The proportion of patients on ‘bridging’ neoadjuvant endocrine therapy (NET), who progress or fail to respond to this treatment, and subsequently require surgery for clinical reasons earlier than anticipated.
2. The proportion of patients, who would normally be offered IBR, undergoing simple mastectomy, who then proceed to have a delayed reconstruction, which could include mixed-methods analysis of psychological impact.

The long-term aim is to audit outcomes in patients undergoing COVID-altered treatment. This national cohort of patients can be interrogated in the future for oncological outcomes, for example:

1. The impact of NET on service provision as surgery is reopened
2. The response to NET, in a cohort of patients who would ordinarily have surgery
3. Impact of COVID-altered management on local and distant recurrence

## 2 Methods and analysis

This is a multi-centre collaborative study of consecutive patients with breast cancer undergoing MDT treatment decisions during the acute and recovery phases of the COVID-19 pandemic. The recruitment period is from the 16th March 2020 (commencement of social distancing recommendations in UK) until the local recommencement of routine breast cancer management as gauged by the availability of pre-COVID services and facilities are determined. It is anticipated that most units will resume standard practice at a similar time point.

### 2.1 Patient inclusion and exclusion criteria

Inclusion criteria

• New diagnosis of non-invasive / invasive primary breast cancer (pathological classification B5a, B5b, B5c [18])
• AND treatment during the COVID-19 pandemic (from the 16th March 2020 until the recovery phase of the pandemic is established)

Exclusion criteria:

• recurrent or metastatic breast cancer
• diagnosis of benign breast disease (i.e. no malignancy found)
• patients undergoing primarily symmetrising and risk-reducing surgery
• surgery for indeterminate or suspicious (B3/B4) lesions detected on core biopsy

### 2.2 Study design

All breast cancer units in the UK and Europe are eligible to participate. Study information is available on the website bmapc.org, which hosts the collaborator recruitment sign-up process. The UK Association of Breast Surgery has endorsed the study and encouraged all members to participate.

This is a multi-centre collaborative audit. Collaborative study design is a well-tested method of delivery of high-quality cohort studies in the UK [14], [19], [20] with the capacity to generate meaningful large scale data with the potential to inform or change clinical practice. There have been several successful trainee collaborative studies related to breast surgery [21], [22], [23], [24].

### 2.3 Data collection

All eligible consecutive patients will be identified prospectively by the local participating clinical teams via the documentation from local multidisciplinary team (MDT) meetings. It is assumed that standard or COVID-altered treatment pathways for each patient will be clearly recorded in the MDT records, as per ABS guidelines. Prospective data entry is preferred, following the MDT meeting. It is accepted that in some centres, for example with a high burden of COVID-19 patients, retrospective data collection will be required.

Data collection will occur in accordance with Caldicott II principles. All data will be recorded in an anonymised format using a unique alphanumeric study identification number. A secure record of NHS numbers and corresponding REDCap ID will be kept locally to facilitate ‘staged’ data collection and future outcome studies. No patient identifiable data will be collected or recorded centrally for the purpose of the audit. Study data will be collected and managed using REDCap™ electronic data capture tools hosted at The University of Manchester [25], [26]. REDCap™ (Research Electronic Data Capture) is a secure, web-based software platform designed to support data capture for research studies, providing 1) an intuitive interface for validated data capture; 2) audit trails for tracking data manipulation and export procedures; 3) automated export procedures for seamless data downloads to common statistical packages; and 4) procedures for data integration and interoperability with external sources [27].

**Fig. 1:**
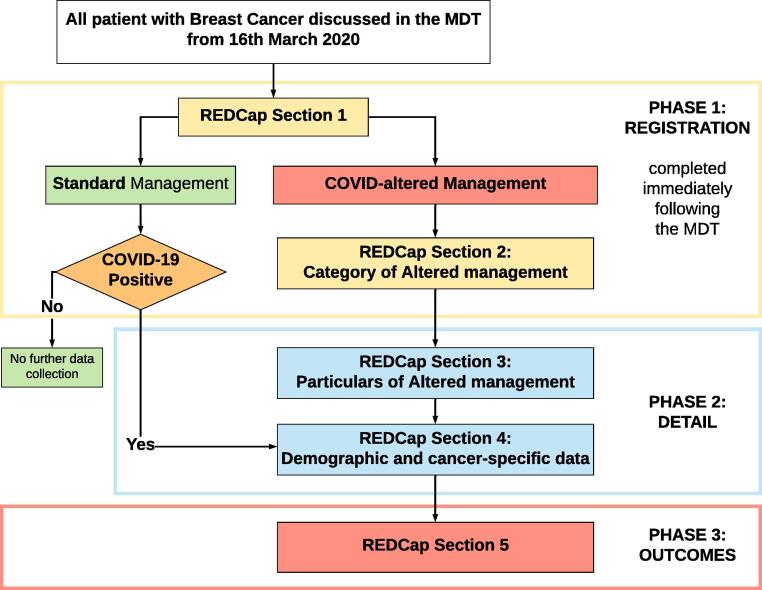
Phases of data collection.

Data collection will be conducted in three phases (Fig. 1).

1. Phase 1 will register patients to the study, document cancer TNM stage, and classify the type of treatment received (standard or COVID-altered).
2. Phase 2 will collect the details of patient demographics, imaging, staging, pathology results and the specifics of the COVID-altered treatment.
3. Phase 3 will collect long-term treatment and outcome data for specific subsets of patients. This phase will be iteratively developed depending on the evolution of the pandemic and the subsequent data collected in phases 1 and 2.

For details of the REDCap Sections 1–5 data collection, please refer to Supplement 1

### 2.4 Data validation and management

For quality assurance purposes, REDCap’s built-in data quality rules will be used to find discrepancies and errors in the project data. In addition, in the lead up to publication, interim analyses will be performed to look for discrepancies in the data, and if identified, the study site will be contacted to validate that record. This is consistent with the quality assurance procedure used in other large collaborative audit projects [19]; however, rather than selecting a random sample to validate, specific cases will be identified.

### 2.5 Data analysis

The study report will be prepared according to the STROBE (Strengthening the Reporting of Observational Studies in Epidemiology) reporting guidelines for observational studies [28]. All data analysis will occur centrally by the B-MAP-C study team with input from a biostatistician. It is anticipated that simple descriptive summary statistics will be calculated for each outcome. Data will be tested for normality and differences between groups using unpaired t-tests, Mann-Whitney U tests and Chi squared tests if appropriate.

## 3 Ethics and dissemination

Ethical approval is not required for this cohort study as it was classified as an audit according to the NHS Health Research Authority online decision tool http://www.hra-decisiontools.org.uk/research/. However, each participating centre is required to register the study locally and obtain local governance approvals prior to commencement of data collection. Data collection will be based on routine clinical data already available to local clinical teams. Collected data will be compared to established standard breast cancer management [29] as well as COVID-specific guidelines published by the ABS [6] and the RCR [7], [10].

Local audit data will be available to individual participating centres at any given time. Participating centres will have ownership of their own data. Results of data analysis will be submitted for publication, the ABS newsletter and a webinar. All data will be presented at national and international conferences, circumstances permitting.

## 4 Discussion

This European study aims to describe the extent of alterations in the management of breast cancer during the COVID-19 pandemic on a patient and population-based level to help us understand the true impact of the pandemic on patient outcomes and the degree to which breast cancer management has been affected. This knowledge will assist us with the long-term planning of service delivery once routine breast cancer management resumes or if another pandemic were to occur. In addition, our findings will be helpful for long-term counselling of patients with breast cancer. Since the outcomes of our study are likely to evolve depending on the progression of the pandemic, we will discuss briefly some of the potential findings.

In the UK, the National Institute for Health and Care Excellence (NICE) guidelines recommend that all women, undergoing a mastectomy, should be offered breast reconstruction, in view of the potential psychological effects associated with the loss of a breast [29]. However, the COVID-19 pandemic has led to suspension of IBR in many countries worldwide due to resource, workforce and safety concerns [30]. We aim to identify this cohort of women that did not have IBR as a result of the pandemic and audit patient-specific outcomes. For example, we could determine the type of reconstruction performed and the duration of waiting time against published guidelines [31], as well as assess the impact on patients’ psychological and mental health [32], [33].

We will audit the outcomes of neoadjuvant endocrine therapy (NET) in a real-world setting. Based on the national guidelines, it is anticipated that significant number of patients with ER + HER2- cancers will have their surgery deferred to minimise the risk of COVID-19 transmission as a result of hospital admission for surgery. There is evidence to suggest that NET may be utilised in post-menopausal women to facilitate breast conservation in 30–70% of women requiring mastectomy at baseline [34], [35], [36]. There is also evidence suggesting its clinical equivalence to neoadjuvant chemotherapy in post-menopausal women with ER positive disease [34]. The use of NET in pre-menopausal women is less well established and will be evaluated in this study.

In the long term, we will have the opportunity to assess the impact of treatment alterations on the rate of disease recurrence and overall patient survival. If breast cancer outcomes following altered management are shown to be comparable to standard treatment, it may prompt future interventional trials.

Finally, we anticipate that the B-MaP-C study will strengthen the collaborative breast cancer research network and will reinforce multidisciplinary links with the medical and radiation oncology research communities, allowing for better collaboration in the future.

## 5 Study status

Data collection has commenced across the UK and Europe, with 59 UK units and 7 European units registered to participate to date. Interim analysis for the ‘acute phase’ of COVID-19 is expected to be undertaken at the end of June 2020.

## 6 Authors’ contributions

RVD designed the study, wrote the initial proposal, provided trainee collaborative expertise.

AC inputted on the study design, drafted the manuscript based on the study proposal, and is part of the audit advisory group.

ROC provided trainee collaborative expertise, advised on the study protocol, and is part of the audit advisory group.

TR provided trainee collaborative expertise, advised on the study protocol, and is part of the audit advisory group.

CK, AG, RIC, were involved in study design, advised on the protocol and are part of the audit steering group.

KH, SMcI, DL, RV, SP, CH, CC, EC, CC, NS, PF were involved in study design, advised on the protocol and are part of the audit advisory group.

BK was involved in study design, advised on the protocol, piloted the RedCap database, and is part of the audit steering group.

## 7 Funding statement

This research did not receive any specific grant from funding agencies in the public, commercial, or not-for-profit sector.
